# Relevance of cycle threshold values in mass screening by reverse-transcription-PCR during COVID-19 pandemic in Belgium: a decision-making support?

**DOI:** 10.2217/fvl-2022-0020

**Published:** 2022-09-16

**Authors:** Patrice Dufour, Henry Paridaens, Jean-Marc Senterre, Jean-Marc Minon

**Affiliations:** ^1^Department of Laboratory Medicine, Centre Hospitalier Régional de la Citadelle, Liège, 4000, Belgium

**Keywords:** COVID-19, epidemiology, mass screening, reverse-transcription PCR

## Abstract

**Aim:** The Belgium’s strategy against COVID-19 was partly based on mass screening. Here, we reported the results observed in a Belgian mass screening center. **Materials & methods:** Between October 2020 and February 2021, 32,089 samples were collected analyzed with reverse-transcription PCR (Thermo Fisher Scientific kits and apparatus). Patients were categorized according to their contagiousness (extrapolated from the cycle threshold [Ct] values and the recommendation of Sciensano). **Results:** We observed association between Ct values and age, with higher Ct observed in extreme age groups (<6 years and >75 years). **Conclusion:** The analysis of the evolution of the contagiousness of these patients tested twice within a 7-day period showed the relevancy of the recommendation edited by Sciensano.

## Plain language summary

The Belgium’s strategy against COVID-19 was partly based on mass screening performed with reverse-transcription PCR analysis performed on nasopharyngeal samples. Reverse-transcription PCR allows the detection of individuals infected by the virus. In addition, it provides semi quantitative results correlated to the quantity of virus present in the patient, these results are difficult to interpret for the patients and numerous practitioners. In our study, we showed that the interpretation of these results should take into account the age of the patients. We also showed that the semi quantitative result observed in a positive patient is not predictive of the evolution of the viral load (quantity of virus present in the organism) in the 7 following days.

In December 2019, China reported the first cases of SARS-CoV-2. Since then, COVID-19 has spread worldwide and today (July 2021), the pandemic is still not under control and has caused the death of more than 3,800,000 people [1]. In October 2020, Belgium was among the countries reporting the highest number of cases per 100,000 inhabitants (during week 44 of 2020, Belgium recorded its highest rate of infection with a mean rate of 1778.2 cases per 100,000 population on a 14-days basis) [1]. To face the pandemic, many countries, including Belgium, implemented mass screening to identify and isolate patient infected by the virus in order to reduce the disease spread. In Belgium, this mass screening is performed by using real-time reverse-transcription PCR (RT-PCR) tests on nasopharyngeal samples collected with adequate swabs.

**Figure 1.**
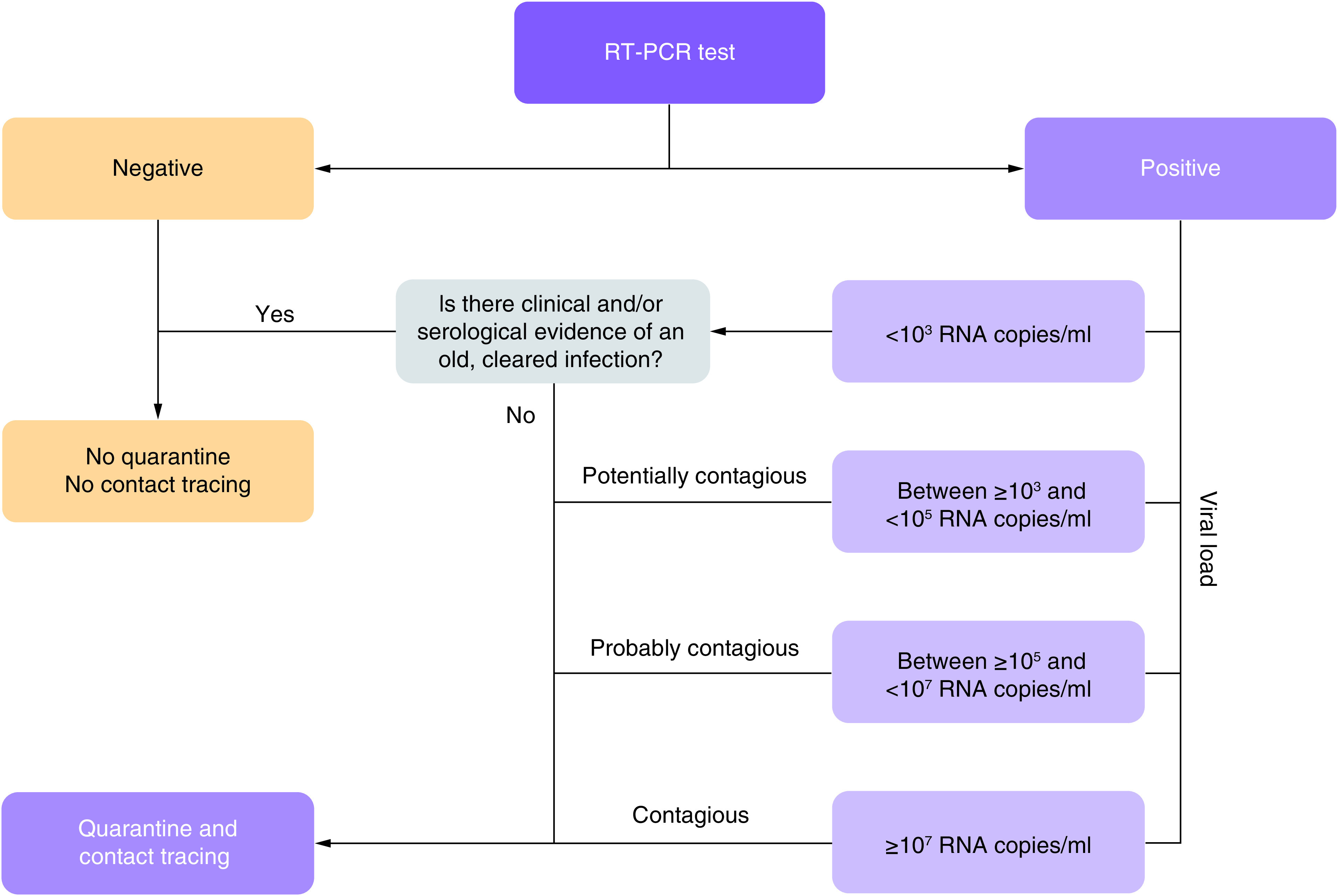
Algorithm for the management of patient tested by reverse-transcription PCR according to the Sciensano’s recommendations.

In the absence of a calibration curve RT-PCR should not be considered as a quantitative analysis [2]. Nevertheless, some authors demonstrated that the cycle threshold (Ct) value of the RT-PCR is inversely correlated with viral load [3] and with virus infectivity [4,5]. Therefore, Sciensano (the public institution serving as reference in the field of public health in Belgium) edited recommendations to interpret Ct values and to evaluate the contagiousness of patients. In addition, the institution sent control materials with known virus concentration to Belgian laboratories to allow them to correlate Ct value measured with their analytical methods and sample viral load. The aim was to propose adapted measures (quarantine, contact tracing, etc.) to manage patient with risk of virus transmission and those who were no longer at risk to transmit the virus [6]. During the study period, quarantine was imposed to individuals with positive PCR test. The duration of quarantine was at least 7 days after the onset of symptoms, release from quarantine required 3 days without fever and the improvement of other symptoms. Quarantine was also imposed to close contacts (cumulative contact of at least 15 min without face mask) for at least 7 days after the last contact (a negative PCR test is then required) and maximum 10 days. Sciensano proposed to report semi-quantitative RT-PCR values to help the clinicians and the epidemiologists but also to identify patient ‘probably not or no longer contagious’ that could avoid quarantine and contact tracing if there is also clinical and/or serological evidence of an old, cleared infection [6]. Figure 1 illustrates the algorithm proposed by Sciensano.

In the present paper, we reported the result of 4 months of COVID-19 mass screening performed in the Department of Laboratory Medicine, Centre Hospitalier Régional de la Citadelle (Liège, Belgium). During this mass screening, we tested ambulatory patients, mainly: symptomatic individuals, individuals in close contacts with positive patients and individuals tested before travel or hospitalization. Data were analyzed in order to compare it with the recommendations edited by Sciensano.

## Materials & methods

### Sample collection

During the pandemic, our laboratory received samples from multiple sources (general practitioners, clinical departments of the hospital, mass screening in collectivities [e.g., schools, nursing homes and so on]). To be consistent, we limited our analysis to the samples collected in our ‘drive-in center’ established in the vicinity of the hospital in order to screen the general population. Nasopharyngeal samples were collected by trained nurses using Biotrading™ liquid amies transport medium (UTM) and flocking sampling swabs. Swabs were conserved in 2 ml of viral transport medium and were transported within 2 h to the laboratory at ambient temperature. Samples were then conserved at 4°C until they were extracted and analyzed within a maximum of 24 h. The sample collection period took place between 22 October 2020 and 26 February 2021. This period corresponds to the second wave of COVID-19 pandemic in Belgium. During this period 32,089 samples were collected and analyzed.

### RT-PCR analysis

The samples (UTM) were opened in a biosafety cabinet class-II and 200 μl of the viral transport medium (VTM) was further processed for viral nucleic acid extraction with a MagMAX Viral/Pathogen kit on KingFisher Flex™ instruments (Thermo Fisher Scientific, Belgium) according to the manufacturer’s protocol.

A 25 μl reaction was then prepared for qualitative detection of SARS-CoV-2 by RT-qPCR utilizing 10 μl of extracted RNA, 6.25 μl of TaqPath 1-Step Multiplex Master Mix (Thermo Fisher Scientific), 1.25 μl of TaqPath COVID-19 Assay Multiplex (Thermo Fisher Scientific) and 7.5 μl of RNAse free water. All oligonucleotides included in the Taq Path COVID-19 Combo Kit were synthetized and provided by Thermo Fisher Scientific. Thermal cycling was performed at 53°C for 10 min for RT followed by 2 min at 95°C and then 40 cycles of 3 s at 95°C and 30 s at 60°C using an Applied Biosystems Quant Studio 5™ real-time PCR, 96-well (Thermo Fisher Scientific). All samples were detected for the *S*, *N* and *ORF1ab* genes. The cut-off threshold (Ct value) for each sample was recorded and samples with Ct value ≤35 (as recommended by the manufacturer) were considered as positive. Each samples PCR was completed within 4 h to avoid RNA degradation. Our laboratory also participated in a multicenter evaluation coordinated by UZ Leuven to harmonize the semi-quantitative reporting of RT-PCR values for SARS-CoV-2 (Rag interpretation and reporting of SARS-CoV-2 PCR results). The control materials consisted in SARS-CoV-2 stock solution of the SARS-CoV-2 BetaCOV/Belgium/GHB-0127/2021 strain from cell culture of clinical samples. Dilutions of 1.10 × 10^8^ copies/ml (1/10 of the undiluted stock) to 1.10 × 10 copies/ml (1/10 of the 10^-7^ dilution) were performed in our laboratory and those dilutions were then tested by the RT-PCR TaqPath COVID-19 assay (Thermo Fisher Scientific) in triplicates. Linear regression lines were thus obtained from these triplicates establishing the relationship between the viral load of the different dilutions and their corresponding Ct for the *S*, *N* and *ORF1ab*-targeted genes.

### Data analysis

Data analysis was performed using Excel 2013 (Microsoft, WA, USA) and RStudio (version 3.4.1; R Project for Statistical Computing). Patients were categorized by age categories (<6; 6–11.9; 12–17.9; 18–74.9 and ≥75 years), These groups were chosen because we hypothesized that these age categories could influence the nurse during the sampling procedure (fear to hurt more fragile individuals). We also categorized individuals according to their presumptive contagiousness. Following the recommendation of Sciensano, individuals with estimated viral load ≥10^7^ RNA copies/ml were considered as contagious, estimated viral load ≥10^5^ and <10^7^ RNA copies/ml was considered as probably contagious, estimated viral load ≥10^3^ and <10^5^ RNA copies/ml as potentially contagious and estimated viral load <10^3^ RNA copies/ml as probably not contagious [6]. These cut-offs were defined by the Sciensano’s group experts according to the data available in the literature (reviewed in Jefferson *et al.*) and the experimental data of viral cultures performed in the Katholieke Universiteit Leuven (Belgium) (Unpublished Data). Ct value measured for the gene *ORF1ab* was used as reference value and we considered Ct ≥ 35 as negative (and not contagious). Comparison of median Ct value between age groups was performed with Kruskal–Wallis test. Comparison of median Ct value between genders was performed with Mann–Whitney test. We also assessed the relation between age and Ct value by computing a second degree polynomial regression model. The significance level was set at p < 0.05.

We also highlighted individuals who got tested more than once during the collection period. If one (or more) of the multiple tests was positive, we considered tests taken less than 8 days before or after the positive test and we evaluated the evolution of contagiousness status during this restrained period.

## Results

**Figure 2.**
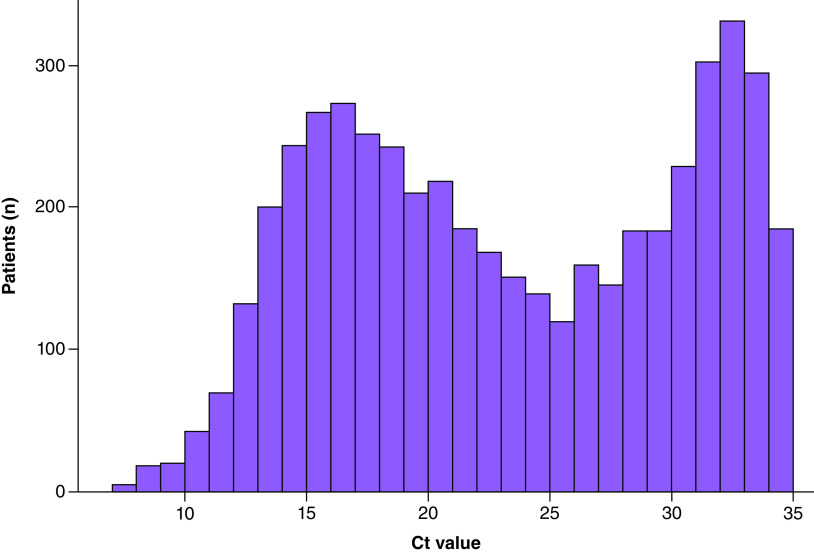
Cycle threshold value distribution among the studied population.

**Figure 3.**
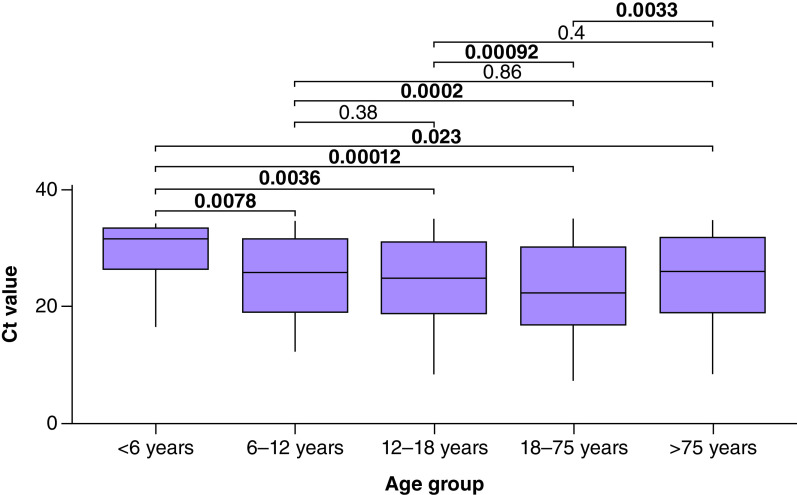
Cycle threshold values according to age group. Pairwise comparisons between groups were performed by using Kruskal–Wallis test. Significant differences were highlighted in bold (p < 0.05).

**Figure 4.**
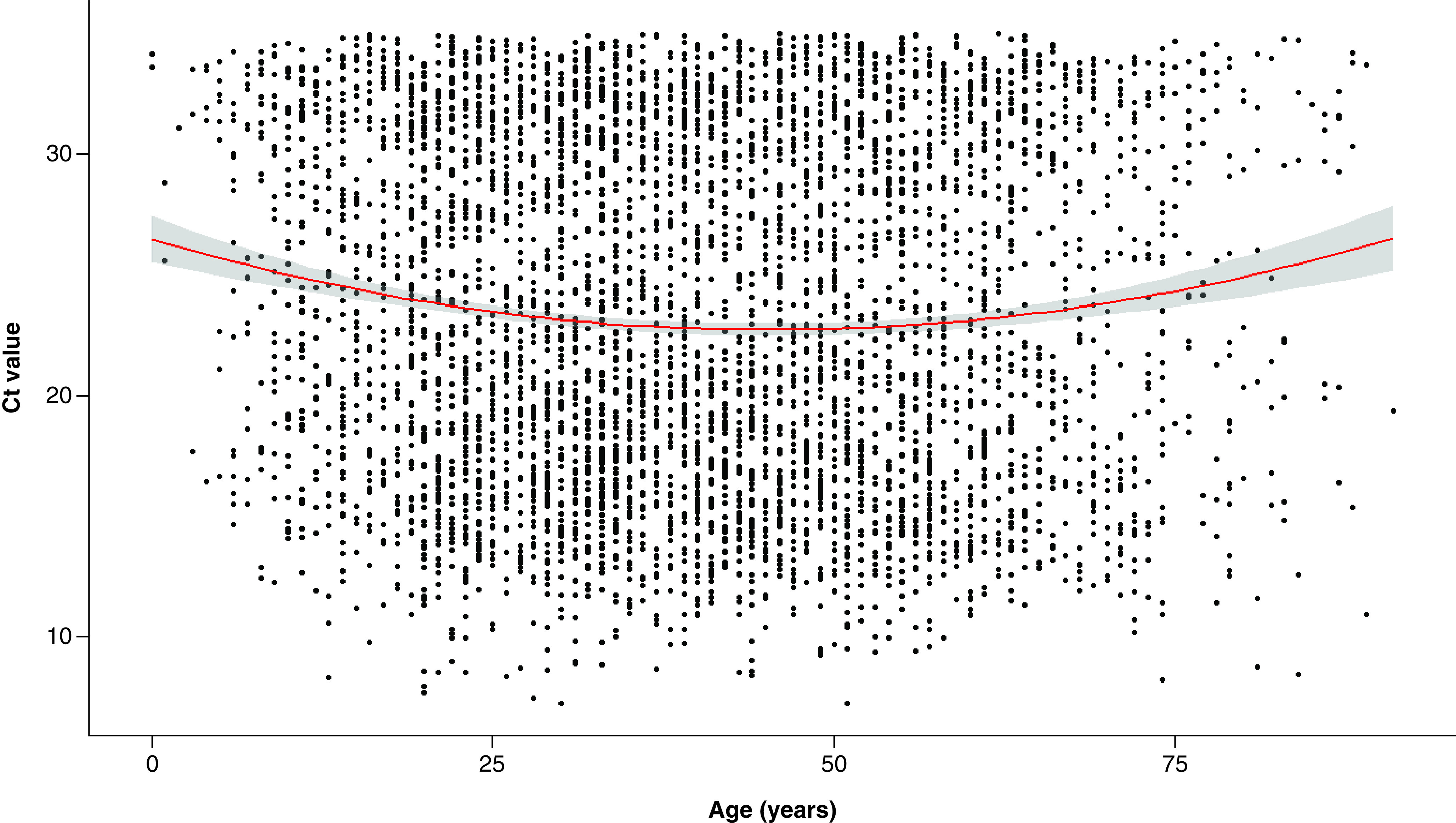
Cycle threshold value distribution according to age. Red represents the curve (95% CI in grey) of the polynomial model computed to assess relation between Ct values and individual age. Ct: Cycle threshold.

Among our population, 16,049 individuals (50.0%) were male and 4958 (15.5%) were tested positive. Among positive patients, the number of women (n = 2488) and men (n = 2470) was similar. Figure 2 illustrates the distribution of Ct values in our positive group. The distribution was bimodal with one peak at Ct = 17 and one at Ct = 32. The median age in our population was 39 years (mean = 39.8; range: 0–98 years). Figure 3 shows boxplot for Ct values according to age group. The median Ct values in <6 years (n = 22, 14 males and 8 females), 6–11.9 years (n = 170, 96 males and 74 females), 12–17.9 years (n = 281, 138 males and 143 females), 18–74.9 years (n = 4374, 2162 males and 2212 females) and >75 years (n = 111, 60 males and 51 females) were 31.5, 25.7, 24.8, 22.3 and 26.0, respectively. Pairwise comparison showed that in one hand, median Ct value in <6 years group was significantly higher than in all other age groups, and on the other hand, the median Ct value in 18–74.9 years was significantly lower than in all other age groups. Figure 4 shows the distribution of the Ct values according to age, this plot also includes the regression line. The equation of the polynomial model was: Ct value = (0.0018 × age^2^) – (0.166 × age) + 26.5. This model is highly significant (p < 0.0001) and shows that minimal Ct values were observed in individuals aged approximately 46 years while higher Ct values were measured in extreme ages. There was no significant difference between median Ct values measured in male (Ct = 22.7) and in female (Ct = 22.6) (p = 0.54), no significant difference was found either when analyses were stratified by age groups.

**Figure 5.**
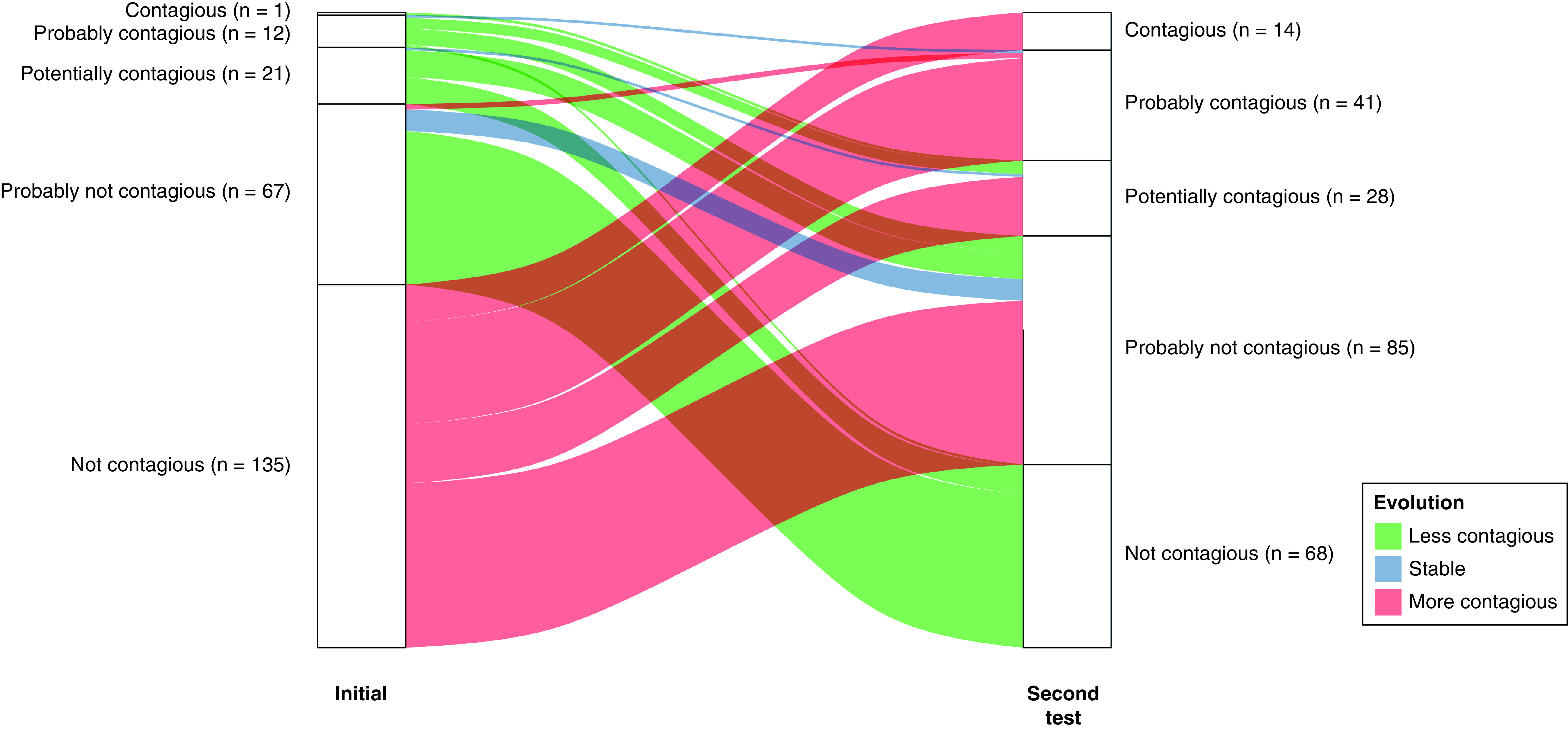
Alluvial plot illustrating the dynamics of contagiousness of patients tested twice within 7 days and with at least one positive test during this 7-day period.

In total, 1860 patients were tested twice within 7 days. Among them, 236 individuals (12.7%) were tested at least once with a Ct value <35. Among these individuals, 137 were more contagious at the time of the second test (age group repartition: <6 years, n = 0; 6–11.9 years, n = 10; 12–17.9 years, n = 14; 18–74.9 years, n = 109; and >75 years, n = 4), 89 were categorized as less contagious (age group repartition: <6 years, n = 2; 6–11.9 years, n = 8; 12–17.9 years, n = 2; 18–74.9 years, n = 75; and >75 years, n = 2) and ten presented the same contagiousness (all were in the age group 18–74.9 years). Among the 137 individuals with an increasing contagiousness, all but two were initially tested as not contagious (Ct >35). Patient contagiousness status at the initial test and at the second test was illustrated in the alluvial plot (Figure 5).

## Discussion

Mass screening of ambulatory patients in the context of the COVID-19 pandemic posed new challenges for clinical biology laboratories. The aim of mass screening is to detect individuals infected by the virus and likely to spread the virus in their environment. Ideally, we have to distinguish a recent infection (the patient is then considered as contagious) from an old infection (the patient is then considered as not contagious and consequently, no further measures are required) [6]. Such task is difficult and should be performed with caution. Indeed, in the case of ambulatory patients, little to no information is known about their symptoms (although, since patients are ambulatory, we can expect that, in the most cases, symptoms were minor to moderate or even absent and that no treatment was given to patient prior to testing) and the delay between the sample collection and the potential symptoms onset. This information can help discriminate between a recent infection from an old one but these data are rarely available for the laboratory. Moreover, in the case of mass screening, numerous patients had no direct contact with their general practitioner before or after the test, which makes the collection of medical information even more difficult. Finally, in the general population, there are many patients infected by SARS-CoV-2 virus that are asymptomatic. A meta-analysis reported that 15.6% of positive individuals are asymptomatic at initial diagnostic among which 51.1% will remain asymptomatic [7]. Virus can be transmitted by droplets, aerosol and conjunctiva and studies showed that viral load of respiratory samples were similar in asymptomatic and symptomatic patients [8,9]. Transmission potential of asymptomatic individuals seems to be lower than those of symptomatic patient but still substantial [8,10]. Therefore, Sciensano’s recommendation to interpret Ct values should be cautious and as general as possible in order to identify and isolate the most contagious patients.

The counterpart of this approach is to impose quarantine and contact tracing at individuals who are no longer at risk of transmitting the virus. Therefore, these recommendations were particularly strenuous for the population and had an important socio-economical impact on the society. This cost was not justified during all the phases of the pandemic. This is why Siensano’s recommendations varied across the pandemic depending on vaccination coverage, virus variant severity or different prevalence according to the season. The recommendations discussed in the present study were implemented during a pandemic wave characterized by a minimal vaccination coverage (during winter 2020–2021, in Belgium, only elderly patients in nursing homes received the first dose of vaccine), absence of virus variants associated with reduced severity and a rise of contaminations because of winter weather. During this period the guidelines of Sciensano were in accordance with those proposed by the Infectious Diseases Society of America [11] which recommends testing symptomatic patients but also asymptomatic patients who were in close contact with infected individuals. This society also recommended performing a second test after a negative one if there is a high suspicion of infection. In China [12], strategies were more drastic: some local governments tested everyone in the area/city in the hope to rule out the virus risk.

Our study allows the comparison of Sciensano’s recommendations with the results of a mass testing center. Sciensano considered patients with viral load above or equaling to 10^5^ RNA copies/ml as contagious or probably contagious notwithstanding the potential evidence of an old infection. For individuals with viral load below 10^5^ RNA copies/ml, Sciensano recommended to objectify an old infection (serologically or with clinical evidence) before considering a patient as not contagious. Our results support this recommendation. Indeed, among the patients initially diagnosed as not contagious, 61 and 22 were tested during the second test as probably not contagious and potentially contagious, respectively. Thereby, we can assume that these patients were at the early stage of the infection and became more contagious a few days later. Moreover, among the patients initially considered as probably not contagious, two individuals were later diagnosed as probably contagious. Again, these patients were probably tested in the early infection and became more contagious a few days later when the infection became more significant. Nevertheless, the cut-offs established by Sciensano should be considered with caution because there is no consensus in the literature.

During the study period, Sciensano also recommended two tests (one on day 1 and one on day 7) in case of ‘high risk contact’ or after traveling in a foreign country. Again, our results highlight the relevance of this recommendation. Indeed, among the individuals tested twice within 7 days, 1769 were initially tested as not contagious and among them, 135 (7.6%) were tested as positive at the second test. These individuals, if they were asymptomatic, could have been ignored and become a vector of virus transmission if one test alone had been recommended.

Sciensano recommended evaluating patient contagiousness according to the number of RNA copies/ml extrapolated from the Ct value measured on suspension from nasopharyngeal swabs [13]. Nevertheless, several authors questioned this method since the quality of this sample is highly dependent on the ability of the operator and the tolerance of the patient [14,15]. We observed higher Ct values in extreme age groups. Rabaan *et al.* [16], reported several pre-analytical (collection technique, type of specimen), analytical (internal control, type of RT-PCR, purity of reagents) and post analytical (interpretation of the results) parameters that can influence the Ct value measured. Nevertheless, all the samples tested in the present study follow the same analytical protocol, with the same materials and the same RT-PCR technique and apparatus. Therefore, we hypothesize that the higher Ct values measured in extreme age groups could be partly explained by collection technique issues. Indeed, although studies showed that higher age was associated with higher risk of a severe clinical presentation of COVID-19 [17], we observed that age groups 6–11.9, 12–17.9 and >75 years had similar median Ct value while the age group 18–74.9 years presented a significantly lower median Ct value and the age group <6 years the highest median Ct value. We hypothesize that operators were less respectful of the correct sampling protocol with youngest and oldest patients in order to reduce inconvenience for patients who seemed more fragile. This could have public health impact since children or older patients could have been wrongly determined as less contagious or not infected by COVID-19 because of preanalytical issues. Other authors assessed the relation between Ct value and age groups. Our results were partly in accordance with the observations of [18] who studied Ct value in a German population between 15 March and 15 September 2020 and showed significantly higher median Ct value in older age groups (80–89 and 90–99 years) but not in younger patients [18]. Contrariwise, Buchan *et al.* [19] performed massive testing in Winsconsin (USA) and reported lower Ct value in the group 80–89 years compared with the others age groups. More studies are required to explore these discrepancies between countries.

Our results should be considered with caution. Indeed, in the region surrounding our testing center, several other institutions participated in the mass testing, and therefore, some patients could take the initial or the second test in another institution than ours. Nevertheless, the number of patients who were tested twice within 7 days in our institution is high (n = 1860) and we see no reason why these individuals presented different characteristics from those that were tested in two different laboratories. Finally, we did not know the clinical history of the patient included in our study, so we were not able to compare the Ct values or the evolution of patient contagiousness with clinical parameters.

## Conclusion

The outbreak of the COVID-19 pandemic led the world in an unprecedented situation in recent history. To face this event and to reduce the impact of the pandemic, authorities had to edit and implement a number of rules and protocols. This study compared these rules to the reality of a laboratory actively participating in the mass screening of the Belgian population and showed the relevancy of the protocols implemented in Belgium during the fall and the winter 2020–2021. Nevertheless, some parameters independent from the SARS-CoV-2 virus itself, like the age of the patient and the potentially related preanalytical issues could have an impact on the quality of the results of the mass testing.

**Summary points**

- During fall and winter 2020–2021, Belgium was among the countries reporting the highest number of cases of COVID-19 per 100,000 inhabitants.
- Between October 2020 and February 2021, 32,089 samples were collected in our ‘drive-in center’ (Liege, Belgium), analyzed by using real-time reverse-transcription PCR (RT-PCR) for SARS-CoV-2 detection and 4958 (15.5%) individuals were tested positive.
- Cycle threshold (Ct) values measured for COVID-19 are lower in middle age group while highest median Ct value was observed in the <6 years group. We hypothesized that this observation is due to sample collection issue.
- Patients were categorized according to their allegedly contagiousness. This contagiousness was determined according to the Ct values and the viral load associated.
- Contagiousness was defined as follows: estimated viral load ≥10^7^ RNA copies/ml was considered as contagious, estimated viral load ≥10^5^ and <10^7^ RNA copies/ml was considered as probably contagious, estimated viral load ≥10^3^ and <10^5^ RNA copies/ml as potentially contagious and estimated viral load <10^3^ RNA copies/ml as probably not contagious.
- Evolution of contagiousness was studied in patients tested twice within 7 days and we showed the relevancy of the recommendation of Sciensano (the public institution serving as reference in the field of public health in Belgium). The aim of Sciensano’s recommendations was to apply quarantine and contact tracing to patients who are contagious or at risk of becoming contagious.
